# Salicylic Acid Modulates Volatile Organic Compound Profiles During CEVd Infection in Tomato Plants

**DOI:** 10.3390/metabo15020102

**Published:** 2025-02-06

**Authors:** Marc Balanzá, Francisco Vázquez-Prol, Ismael Rodrigo, José María Bellés, Francisco Vera-Sirera, Maria Pilar López-Gresa, Purificación Lisón

**Affiliations:** Instituto de Biología Molecular y Celular de Plantas, Universitat Politècnica de València (UPV)-Consejo Superior de Investigaciones Científicas (CSIC), Camino de Vera s/n, 46022 Valencia, Spain; marbaga1@etsiamn.upv.es (M.B.); fravazpr@ibmcp.upv.es (F.V.-P.); irodrig@ibmcp.upv.es (I.R.); jmbelles@btc.upv.es (J.M.B.); fravesi@ibmcp.upv.es (F.V.-S.); mplopez@ceqa.upv.es (M.P.L.-G.)

**Keywords:** salicylic acid, volatile organic compounds (VOCs), tomato plant defence, citrus exocortis viroid (CEVd), metabolomics, gas chromatography–mass spectrometry (GC-MS)

## Abstract

**Background:***Citrus Exocortis Viroid* (CEVd) is a non-coding RNA pathogen capable of infecting a wide range of plant species, despite its lack of protein-coding ability. Viroid infections induce significant alterations in various physiological and biochemical processes, particularly impacting plant metabolism. This study shows the metabolic changes upon viroid infection in tomato plants (*Solanum lycopersicum* var. ‘MoneyMaker’) exhibiting altered levels of salicylic acid (SA), a key signal molecule involved in the plant defence against this pathogen. **Methods:** Transgenic *RNAi_S5H* lines, which have the salicylic acid 5-hydroxylase gene silenced to promote SA accumulation, and *NahG* lines, which overexpress a salicylate hydroxylase to degrade SA into catechol and prevent its accumulation, were used to establish different SA levels in plants, resulting in varying degrees of resistance to viroid infection. The analysis was performed by using gas chromatography–mass spectrometry (GC-MS) to explore the role of volatile organic compounds (VOCs) in plant immunity against this pathogen. **Results:** Our results revealed distinct volatile profiles associated with plant immunity, where *RNAi_S5H*-resistant plants showed significantly enhanced production of monoterpenoids upon viroid infection. Moreover, viroid-susceptible *NahG* plants emitted a broad range of VOCs, whilst viroid-tolerant *RNAi_S5H* plants exhibited less variation in VOC emission. **Conclusions:** This study demonstrates that SA levels significantly influence metabolic responses and immunity in tomato plants infected by CEVd. The identification of differential emitted VOCs upon CEVd infection could allow the development of biomarkers for disease or strategies for disease control.

## 1. Introduction

Plants are exposed to a variety of stresses that can significantly impact their growth, development, and overall fitness. These stresses are classified as abiotic, caused by environmental factors such as drought, extreme temperature, salinity, and shading [1], or biotic, resulting from pathogen and herbivore attacks [2]. In response to these challenges, plants have evolved complex and highly regulated defence mechanisms.

In this context, phytohormones serve as critical signalling molecules in the plant’s response to various stresses. Salicylic acid (SA) plays a central role in plant defence against biotrophic and hemibiotrophic pathogens [2]. The generation of transgenic plants overexpressing the bacterial *NahG* gene, which prevents SA accumulation by converting it to catechol, was fundamental in elucidating the pivotal role of SA in plant defence. These *NahG* plants were unable to establish effective defence mechanisms [3,4]. Additionally, the exogenous application of SA has been shown to induce the accumulation of pathogenesis-related (PR) proteins, which confer protective effects in infected plants [5], further corroborating the essential role of SA in plant defence.

SA accumulates in varying quantities depending on the specific plant–pathogen system and can also be converted into some derivates like gentisic acid (GA, 2,5-dihydroxybenzoic acid), a 5-hydroxylated derivate, that dramatically accumulates upon *Citrus Exocortis Viroid* (CEVd) infection, inducing specific defence proteins not triggered by SA, suggesting distinct roles of SA and GA in plant defence responses [6]. The hydroxylation of SA to produce GA is catalyzed by the S5H (salicylate 5-hydroxylase) enzyme in both *Arabidopsis thaliana* [7,8] and tomato plants [9]. Notably, *RNAi*_*S5H*-silenced tomato plants, which accumulate higher levels of SA, display a reduced size, early senescence, and hypersusceptibility to the necrotrophic fungus *Botrytis cinerea*, while exhibiting resistance to biotrophic pathogens such as *Pseudomonas syringae* or CEVd [9].

Viroids are the smallest known plant pathogens, consisting of a small circular single-strand RNA molecule (250–400 nucleotides) with a highly stable secondary structure and lacking any envelope or protein capsid [10,11]. Unable to encode any proteins, they parasitize the transcriptional machinery of the host cell, depending on their RNA polymerases and processing enzymes for replication [12]. Among these pathogens, CEVd is notable for infecting various agronomic plant species, including tomato (*Solanum lycopersicum*). Infected plants exhibit symptoms such as bark scaling and stunted growth. CEVd is one of the most extensively studied viroids due to its significant impact on agricultural production [11,13,14]. The role of SA in plant–viroid interactions is well established. For instance, *NahG* tomato plants, which are impaired in SA accumulation, exhibit enhanced susceptibility to CEVd [15], while transgenic *RNAi_S5H* lines with increased SA levels demonstrate greater resistance to this pathogen [9]. Moreover, treatments with BTH, a SA functional analogue, have been described to reverse the enhanced susceptibility of NahG plants to CEVd [15].

Beyond SA, plants produce volatile organic compounds (VOCs) in response to biotic stresses. These VOCs play a dual role since they activate defence responses both in the plant’s own distal tissues and in neighbouring plants, thereby mitigating potential infections [16,17,18,19]. VOCs include a wide range of chemical structures including terpenoids, phenylpropanoids–benzenoids, and derivatives of fatty and amino acids [20,21,22]. These compounds are characterized by their low molecular weight, high vapour pressure, and capacity to cross membranes and be released into the atmosphere [23]. In this context, VOCs have emerged as key players in plant defence, functioning as direct antimicrobial agents, signalling molecules, or attractants for beneficial organisms that aid in controlling pathogen spread [24,25]. Particularly, tomato plants accumulate various VOCs in response to pathogen attack [26,27], and the defensive roles of several of these compounds have been demonstrated [28,29,30,31]. Due to their physicochemical properties, this type of compound has classically been analyzed using GC-MS (gas chromatography–mass spectrometry), a powerful analytical technique that combines the separation capabilities of gas chromatography with the identification and quantification precision of mass spectrometry. GC-MS is particularly well suited for volatile and semi-volatile compounds, allowing for an accurate characterization of complex mixtures.

The aim of this study is to characterize the volatile profile of tomato plants in response to CEVd infection and to investigate the potential role of SA in modulating this volatilome. To achieve this, an untargeted metabolomics approach will be employed to identify differentially accumulated VOCs across genotypes with varying SA levels and viroid symptomatology: *NahG*, *RNAi_S5H*, and the parental ‘MoneyMaker’ (MM). This analysis will be conducted under both CEVd-infected and mock-treated conditions.

## 2. Materials and Methods

### 2.1. Plant Material, Viroid Inoculation, and Exogenous Application of VOCs

Seeds from tomato (*Solanum lycopersicum* cultivar MoneyMaker), transgenic NahG tomato plants overexpressing the bacterial SA hydroxylase transgene [3], and *RNAi_S5H*-silenced plants (RNAi_S5H) [9] were used in this study.

To infect tomato plants with CEVd, cotyledons from the genotypes indicated were inoculated with dimeric viroid transcripts by mechanical or agro-infiltration methods, following Prol et al. [32]. The agrobacterium culture, grown overnight, was diluted with an infiltration buffer (0.01 M MES, 0.01 M MgCl_2_) to an optical density of 1 at 600 nm and infiltrated into the abaxial side of a cotyledon using a syringe without a needle. Systemic leaf samples and height measurements were collected 21 days post-inoculation.

### 2.2. Gas Chromatography–Mass Spectrometry (GC-MS)

Metabolite analyses via gas chromatography–mass spectrometry (GC-MS) were performed as previously described [26]. In brief, 100 mg of frozen leaf tissue was placed in a 10 mL headspace screw-cap vial. The sample was mixed with 1 mL of a saturated CaCl_2_ solution, and 150 µL of 750 mM EDTA (pH adjusted to 7.5 with NaOH) was added. The mixture was gently stirred and sonicated for 5 min. Then, it was analyzed using GC-MS with an Agilent 8860 GC coupled with the 5977B GC/MSD system (both manufactured by Agilent Technologies, Santa Clara, CA, USA).

Samples initially stored at −80 °C were thawed at 50 °C for 10 min prior to extraction. Metabolites were extracted using a 65 μm DVB/PDMS SPME fibre (Supelco; Bellefonte, PA, USA) for 20 min and then analyzed by GC-MS, equipped with a DB-5 ms fused silica capillary column (60 m length, 0.25 mm diameter, 1 µm film thickness). The oven temperature programme started at 40 °C (held for 2 min), increased at a rate of 5 °C per minute to reach 250 °C, and was maintained at this final temperature for 5 min. Helium was used as the carrier gas at a constant flow rate of 1.3 mL/min. The mass spectrometer operated in electron ionization mode at 70 eV, with the ion source temperature set to 230 °C and a continuous scan range from *m*/*z* 35 to 250.

### 2.3. Normalization of Ion (m/z) Profiles Across Genotypes

To compare the volatilome of *NahG* vs. *RNAi_S5H* genotypes in CEVd-infected conditions, ion levels were normalized across genotypes using a global baseline average from mock samples, which was adjusted by a baseline factor specific to each genotype. This process involved the following steps: (I) Calculation of a Reference Baseline: The average ion level across all genotypes under mock (control) conditions was computed to serve as a common baseline reference (global mock average). This average was derived as follows:

$$Global\;Mock\;Average=\frac{\sum_{i=1}^{n}Mock\;level\;of\;Genotype}{n}$$

where n represents the number of samples.

(II) Baseline Adjustment for Each Genotype: For each genotype, the relationship between its individual mock level and the global mock average was determined. This ratio, referred to as the “baseline adjustment factor”, was calculated as follows:

$$Baseline\;Adjustment\;Factor=\frac{Mock\;level\;of{Genotype}_{i}}{Global\;Mock\;Average}$$

(III) Normalization of Treated Samples: Ion levels of treated (CEVd-infected) samples were normalized by dividing their raw values by the corresponding baseline adjustment factor of their genotype. The normalized volatile level for treated samples was expressed as follows:

$$Normalized\;Treated\;Level=\frac{Raw\;Treated\;Level}{Baseline\;Adjustment\;Factor}$$

This normalization ensured that differences in volatile levels across genotypes under mock conditions did not confound the evaluation of treatment effects.

### 2.4. Data Analysis

Chromatogram alignment and quantification of each mass spectral feature were performed using MetAlign software 3.0. The resulting dataset was subsequently processed, analyzed, and interpreted through a Principal Component Analysis (PCA) using MetaboAnalyst 6.0. PCA was performed by first normalizing and scaling the data (Pareto) to reduce variability while retaining meaningful differences. A covariance matrix was calculated to identify patterns of variance, and the data were projected onto principal components (PCs) that capture the majority of the dataset variance. The first two PCs were used to generate a score plot, highlighting clustering patterns between genotypes and treatments, while a loading plot identified key VOCs contributing to the observed differences. Peaks and mass spectra of interest were further analyzed using MassHunter software 10.0 (Agilent). Metabolite identification was conducted by comparing mass spectra with the NIST 07 Mass Spectral Library and validated by comparing both mass spectra and retention times with commercial standards. Statistical analyses were conducted using R-studio, with a significance threshold set at *p* ≤ 0.05 and Origin (Pro), Version 2024, OriginLab Corporation, Northampton, MA, USA.

## 3. Results

### 3.1. Impact of Salicylic Acid Accumulation on Symptom Development and Metabolic Responses in CEVd-Infected Tomato Plants

**Figure 1:**
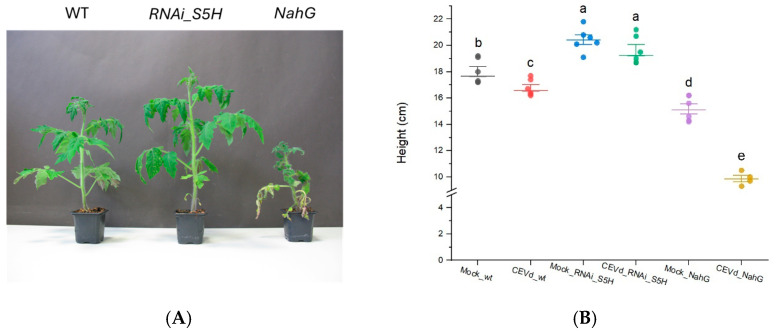
Disease development of representative MoneyMaker (WT), *NahG*, and *RNAi_S5H* tomato plants 21 days after CEVd infection. (**A**) Phenotype of CEVd-infected tomato plants. (**B**) Height of mock-treated and CEVd-infected tomato plants. Error bars represent the standard error of the mean. The letters show the grouping information using Tukey’s range test (one-way ANOVA method) with a significance level of 5% (*p*-value < 0.05).

To better understand the changes in plants during viroid pathogenesis, various approaches have focused on the differential expression of genes related to defensive compounds [2]. Recent studies have highlighted the critical role of salicylic acid (SA) in plant defence and its impact on viroid pathogenesis, where SA concentration determines plant resistance. Particularly, *NahG* tomato plants, unable to accumulate SA, display increased susceptibility to CEVd [15], whereas *RNAi_S5H* transgenic lines with higher SA levels show enhanced resistance to this pathogen [9]. To study the metabolic changes associated with CEVd tolerance and susceptibility, plants corresponding to the three genotypes (WT, *NahG*, and *RNAi_S5H*) were infected with the viroid. As previously reported, *NahG* plants exhibited enhanced susceptibility to CEVd, whereas transgenic *RNAi_S5H* lines showed increased resistance, confirming the essential role of SA in viroid pathogenesis. The heightened susceptibility of *NahG* plants was evident both in the development of symptoms such as epinasty, roughness, and chlorosis (Figure 1A), as well as in compromised growth, reflected by reduced plant height (Figure 1B). In contrast, *RNAi_S5H* plants showed no viroid symptoms or significant height reduction.

Among the secondary metabolites involved in plant immunity, VOCs have attracted significant interest due to their role in activating defence responses in neighbouring plants. Metabolomics studies have been performed to identify VOCs differentially accumulated in response to different biotic stresses [26,33]. However, the volatilome of tomato plants in response to CEVd infection remains unexplored, and the potential role of these volatiles in viroid pathogenesis is yet to be elucidated.

**Figure 2:**
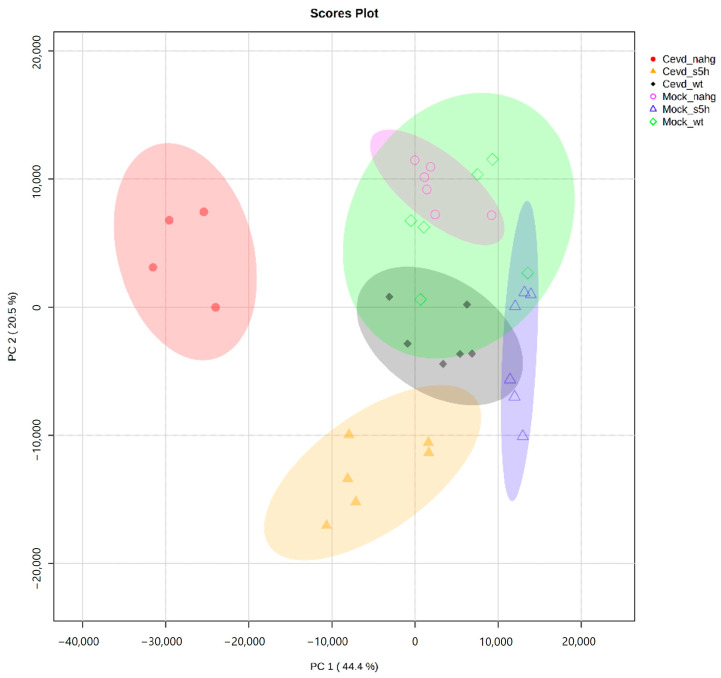
Principal Component Analysis (PCA) score plot based on the whole array of the mass spectra within an *m*/*z* range from 35 to 250 based on Pareto scaling. Samples included *NahG*, RNAi_S5H, and the genetic background ‘MoneyMaker’ (WT) plants, either infected with CEVd, including CEVd_*NahG* (full red circles), *CEVd_S5H* (full orange triangles), or CEVd_WT (full dark-green diamonds), or mock non-inoculated with Mock_*NahG* (open pink circles), Mock_*S5H* (open blue triangles), or Mock_WT (open green diamonds).

To investigate the volatiles involved in the tomato–CEVd interaction, an untargeted metabolomics analysis using GC-MS was conducted on leaves of *NahG*, *RNAi_S5H*, and the corresponding genetic background ‘MoneyMaker’ (WT) plants, either infected or not with CEVd. The Principal Component Analysis (PCA) of the VOC profiles from both CEVd-infected and mock-treated *NahG*, *RNAi_S5H*, and wild-type (WT) tomato plants revealed distinct clustering patterns (Figure 2). The first two principal components accounted for most of the variance, contributing 44.4% (PC1) and 20.5% (PC2), respectively.

The CEVd-infected *NahG* plants (full red circles in Figure 2) formed a distinct cluster on the negative side of the PC1 axis, indicating that this SA-deficient genotype exhibited a specific volatilome profile during CEVd infection. In contrast, CEVd-infected *RNAi_S5H* plants (full orange triangles), which accumulate higher levels of SA, clustered separately along the negative side of the PC2 axis. Since PC2 accounts for a smaller proportion of the total variance, the separation along this axis suggests that these plants undergo fewer changes in VOC profiles, which is indicative of a more controlled metabolic response. CEVd-infected wild-type plants (full dark-green diamonds) grouped closely near the centre-right of the plot, suggesting a lower metabolic response to CEVd infection. Furthermore, mock-treated plants formed overlapping but distinct clusters, occupying central positions while remaining clearly separated from their CEVd-infected counterparts.

Our results suggest that specific VOCs may serve as potential biomarkers for pathogen response, highlighting the distinct defence strategies employed by genotypes with varying levels of tolerance to CEVd response. This underscores the role of these volatiles in plant immunity and the importance of understanding genotype-specific mechanisms for developing targeted resistance strategies.

### 3.2. Salicylic Acid Accumulation Modulate Volatile Profile in CEVd-Infected Tomato Plants

To further investigate the specific metabolites contributing to the distinct metabolic shifts observed in the PCA, a volcano plot analysis was conducted to identify differentially accumulated VOCs between CEVd-infected and mock-treated plants across the *NahG*, *RNAi_S5H*, and wild-type genotypes (Supplemental Figure S1). This analysis enabled the visualization of VOCs that were statistically up- or down-accumulated in response to CEVd infection in each genotype, based on both fold change (FC) and statistical significance (*p*-value).

The volcano plot analysis (Figure S1A) identified specific volatiles significantly accumulated in CEVd-infected ‘MoneyMaker’ tomato plants, with a log 2 FC (CEVd infected/mock) ≥ 1 and ≤−1. Among these, an aldehyde derived from fatty acids such as (E)-2-octenal, the monoterpene derivative *p*-cymene, and various benzenoids, including guaiacol and methyl salicylate (MeSA), were identified (Table 1). In addition, the accumulation of some VOCs decreased upon CEVd infection, including the monoterpenoids linalool and (*Z*)-2-caren-4-ol, and the organic compound malonamic acid. All compounds were unequivocally identified by comparison with pure standards, except for three VOCs (malonamic acid, (*Z*)-2-caren-4-ol, and (*E*,*E*)-Cosmene), which were tentatively assigned based on their high degree of mass spectral scores (match score > 900).

**Figure 3:**
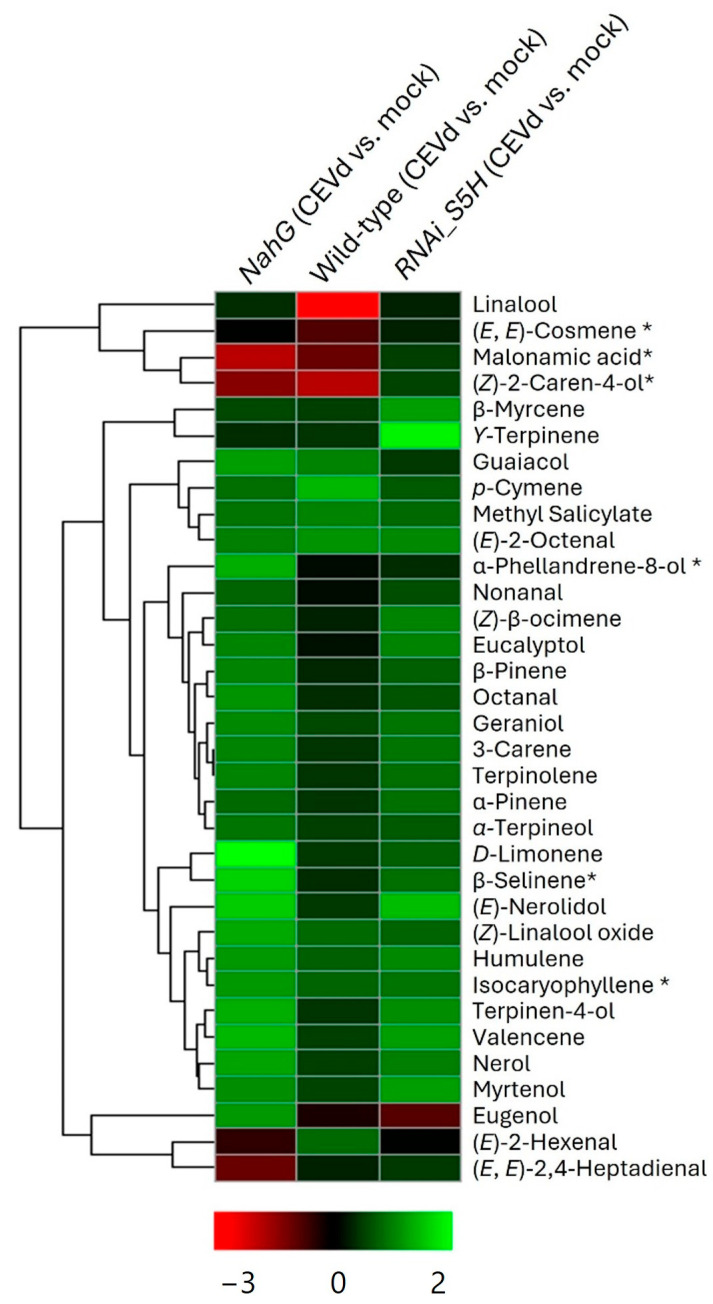
Hierarchical cluster heatmap of the VOCs in CEVd-infected tomato plants across genotypes. The log2-transformed ratios are represented as a heatmap according to the scale below. Red corresponds to higher values; green denotes lower values. Column 1 represents the ratios of the VOCs accumulated by CEVd-infected *NahG* tomato plants versus the mock-inoculated plants. Column 2 represents the ratios of the VOCs accumulated by CEVd-infected WT tomato plants versus the mock-inoculated plants. Column 3 represents the ratios of the VOCs accumulated by CEVd-infected *RNAi_S5H* tomato plants versus the mock-inoculated plants. * Tentative identification based on mass spectrum.

To identify and cluster the compounds associated with plant response to CEVd infection in the three genotypes, a heatmap analysis was conducted (Figure 3). This analysis illustrated the differential accumulation of VOCs across the three genotypes (*NahG*, *RNAi_S5H*, and wild type). The clustering patterns revealed genotype-specific VOC profiles, providing valuable insights into the metabolic pathways either activated or suppressed in response to CEVd infection, highlighting their potential roles in tolerance and susceptibility.

The volcano plot analysis for CEVd-infected *NahG* tomato plants compared to mock-inoculated wild-type plants (Figure S1B) revealed several volatiles that were statistically over-accumulated in these susceptible plants (green colours in column 1 of Figure 3). This so-called “aroma of death” included a diverse range of VOCs including menthane monoterpenes such as D-limonene and terpinolene; menthane monoterpenoids such as α-terpinen-4-ol and α-phellandren-8-ol; acyclic monoterpenoids including nerol and geraniol; cyclic monoterpenoids such as linalool oxide; bicyclic monoterpenes such as β-pinene and 3-carene; sesquiterpenoids like α-humulene and valencene; aldehydes derived from fatty acids like octanal; and benzenoids including guaiacol. These results observed in *NahG* tomato plants suggest a compensatory defence mechanism in response to the absence of salicylic acid (SA). Conversely, transgenic *NahG* tomato plants exhibited reduced accumulation of the aldehydes (*E*)-2-hexenal and (*E*,*E*)-2,4-heptadienal, malonamic acid, and the monoterpenoid (*Z*)-2-Caren-4-ol in response to CEVd infection (red colours in column 1 of Figure 3). These results appear to indicate that SA is involved in their biosynthesis or induction during CEVd infection. These compounds are known for their antimicrobial and signalling roles [34,35], and their diminished levels may reflect a weakened defence response in the absence of SA. This highlights the role of SA in regulating key metabolic pathways involved in plant defence against pathogens.

On the other hand, the specific overproduction of the monoterpenes β-myrcene and α-terpinen-4-ol in CEVd-infected *RNAi_S5H* tomato plants (Figure S1C) may suggest that their biosynthesis is regulated in an SA-dependent manner. The characteristic “aroma of tolerance” (light green colours in column 3 of Figure 3) also includes menthane monoterpenoids (eucalyptol, terpinolene, and ϒ-terpinene), two acyclic monoterpenoids isomers (nerol and geraniol), a bicyclic monoterpenoid (myrtenol), and a sesquiterpene (α-humulene). In contrast, the levels of two benzenoids (eugenol and guaiacol), an acyclic monoterpenoid (*p*-cymene), and a fatty aldehyde derivative (nonanal) were reduced in CEVd-infected *RNAi_S5H* plants (column 3 of Figure 3).

In response to CEVd infection, *NahG* plants, which are deficient in SA accumulation, exhibited significantly higher accumulation of several VOCs. In contrast, *RNAi_S5H* plants, which accumulate higher levels of SA, responded to CEVd infection in a more measured manner, with fewer compounds showing significant fold changes compared to *NahG* plants. The controlled response observed in *RNAi_S5H* plants highlights the regulatory role of SA in modulating volatile accumulation and suggests that plants with sufficient SA levels may rely on more targeted or efficient defence pathways, avoiding the overproduction of volatiles observed in *NahG* plants.

Additionally, Supplementary Table S1 provides a comprehensive catalogue of VOCs significantly altered in CEVd-infected tomato plants compared to mock-treated controls across all genotypes. This detailed analysis highlights the range of VOCs associated with the tomato–CEVd interaction, offering valuable insights into genotype-specific and general responses to infection.

### 3.3. Volatile Organic Compounds as Potential Markers of Tolerance and Susceptibility in CEVd-Infected Plants

**Figure 4:**
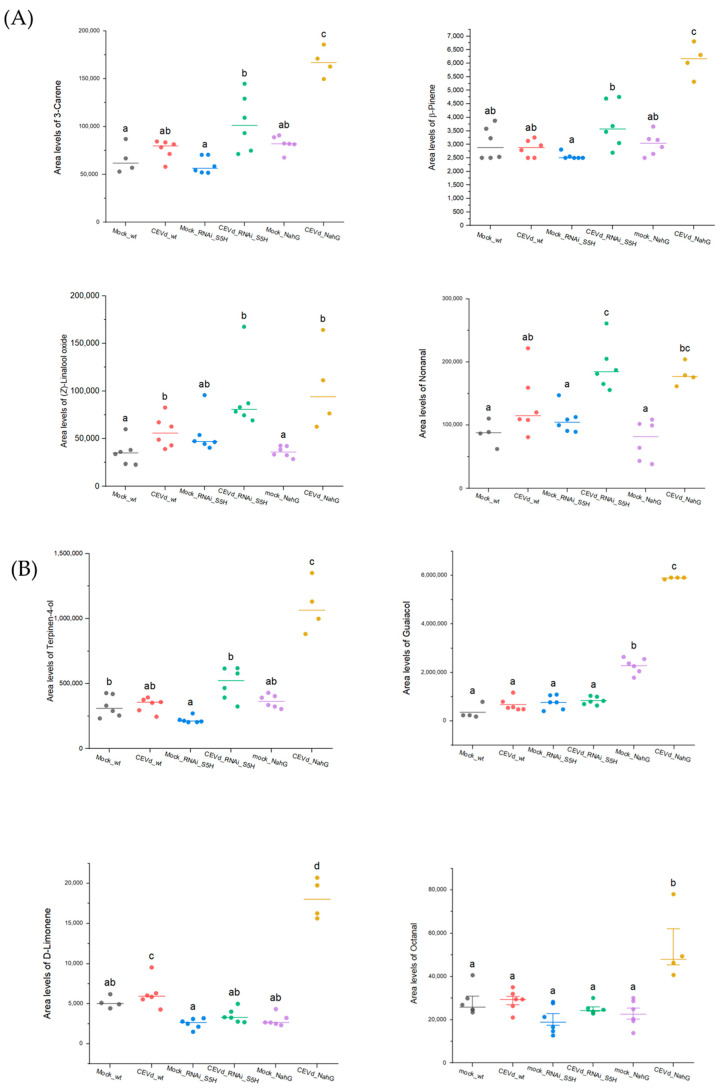

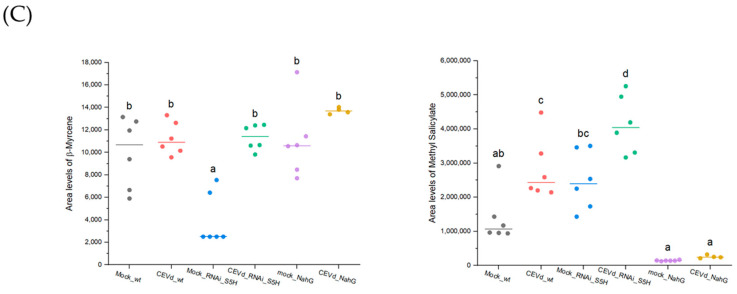
Relative accumulation levels of selected VOCs in tomato plants of different genotypes (WT, RNAi_S5H, and *NahG*) after 21 days of CEVd infection. (**A**) Relative quantification of nonanal, α-pinene, 3-carene, and α-terpinene, characteristic of both the aroma of death and resistance; (**B**) relative quantification of linalool oxide, octanal, guaiacol, and D-limonene, specific to the scent of death; and (**C**) relative quantification of β-myrcene and methyl salicylate, specific to the aroma of resistance. The accumulation levels were quantified based on the peak ion area corresponding to the VOCs from plants inoculated without the pathogen (mock) and inoculated with the CEVd pathogen. The letters show the grouping information using Tukey’s range test (one-way ANOVA method) with a significance level of 5% (*p*-value < 0.05).

To validate the metabolomics analysis, the relative quantification of some discriminant volatiles representative of both the aroma of death and tolerance was conducted. Similar trends were observed for the cyclic monoterpenes β-pinene and 3-carene, the monoterpenoid linalool oxide, and the aldehyde fatty acid derivative nonanal which were found to be common to the aroma of death and tolerance (Figure 4A). The relative quantification of the four VOCs shows a consistent pattern of elevated levels in both *NahG* and *RNAi_S5H* tomato plants upon CEVd infection. This consistent accumulation across genotypes may indicate that these VOCs are part of a broader, SA-independent metabolic response to CEVd infection. Their production could be driven by alternative signalling pathways, such as those involving ethylene, which has also been implicated in plant responses to viroid infections [32]. Alternatively, this response might reflect a general stress-induced increase in VOC production to mitigate pathogen impact or signal neighbouring plants. Further analysis is needed to determine whether these compounds play a direct role in the defence against CEVd or if their accumulation is a byproduct of disrupted metabolic pathways in infected plants.

In turn, the monoterpenoid α-terpinene-4-ol, along with the monoterpene D-limonene, the benzenoid guaiacol, and the fatty aldehyde octanal, were specifically associated with the hypersusceptible *NahG*-infected plants. These VOCs are shared components of the so-called “aroma of death”, suggesting that their production may be associated with symptom development and could therefore be used as biomarkers for viroid infections. Particularly, the monoterpene D-limonene was also found to be over-accumulated in infected wild-type tomato plants, whilst terpinene-4-ol was statistically over-accumulated in *RNAi_S5H*-infected plants when compared with the corresponding non-infected control plants (Figure 4B).

Finally, the linear monoterpene β-myrcene and the phenolic methyl salicylate could be proposed as key components of the “aroma of tolerance” against CEVd, as they are significantly induced exclusively in *RNAi_S5H* plants but not in *NahG* plants (Figure 4C). This highlights their potential role as specific markers of salicylic acid-associated resistance pathways in response to CEVd infection.

**Figure 5:**
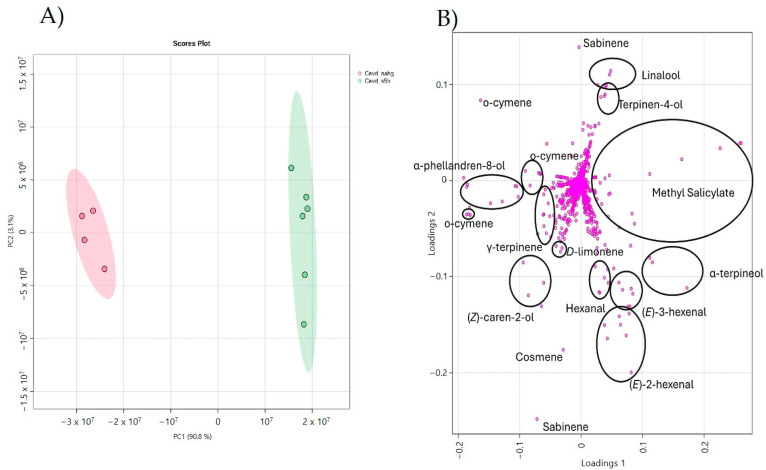
(**A**) Principal Component Analysis (PCA) score plot based on the normalized array of the mass spectra within an m/z range from 35 to 250 based on Pareto scaling. CEVd-infected *NahG* (red) and *RNAi_S5H* (green). (**B**) Loading plot derived from Principal Component Analysis with ion–volatile association.

To further investigate the volatiles associated with both the “tolerance” and “death” aromas, a PCA was performed with data corresponding to CEVd-infected *NahG* plants and CEVd-infected *RNAi_S5H* plants. To reduce potential basal effects introduced by transgenesis, the data were normalized as outlined in the Section 2. As Figure 5A shows, PCA clearly separated the two genotypes upon CEVd infection.

Notably, analysis of the loading plot identified the VOCs that contributed most to the observed variance. In *RNAi_S5H* plants, which exhibit tolerance to CEVd infection, specific monoterpenes and fatty aldehyde derivatives were highly representative. These include terpinene-4-ol, linalool, α-terpineol, the benzenoid methyl salicylate (MeSA), and the fatty aldehydes (*E*)-3-hexenal, (*E*)-2-hexenal, and hexanal. Conversely, VOCs associated with the “aroma of death” predominantly included monoterpenoids and monoterpenes, such as α-phellandren-8-ol, (*Z*)-caren-2-ol, ϒ-terpinene, o-cymene, (*E*,*E*)-cosmene, D-limonene, and sabinene. These findings suggest that two distinct metabolic pathways are involved in CEVd pathogenesis, leading to either tolerance or susceptibility to viroid infection.

## 4. Discussion

Our study highlights the distinct symptomatic (Figure 1) and metabolic (Figure 2) responses to *Citrus Exocortis Viroid* (CEVd) infection of different tomato genotypes with varying levels of SA content: wild-type, SA-deficient (*NahG*), and SA-accumulating (*RNAi_S5H*). Our results emphasize the role of VOCs as potential markers of plant defence, providing critical insights into the influence of SA on plant–pathogen interactions and metabolic reprogramming. These findings align with previous research demonstrating the pivotal role of SA in systemic acquired resistance (SAR) against biotrophic and hemibiotrophic pathogens [5,36]. Moreover, they underscore the potential of SA-mediated pathways to enhance defence responses while mitigating extensive metabolic shifts, offering promising strategies to improve plant resilience against pathogens.

Within the *NahG* genotype, which is unable to accumulate SA due to its conversion to catechol [37], a broad over-accumulation of VOCs was observed upon CEVd infection. This included significant increases in terpene compounds, various sesquiterpenes, aldehydes derived from fatty acids, and benzenoids (Figure S1B, Figure 3, column 1). These results suggest that *NahG* plants appear to be activating an alternative defence mechanism in response to the absence of SA since some of these VOCs have been described to have a direct or indirect role in plant defence [38,39]. Particularly, monoterpenoids have been described to play a role in tomato defence against bacterial infection. Specifically, α-terpineol induces stomatal closure in an SA-independent manner and enhances bacterial resistance in tomato plants [30]. Moreover, JA and ethylene pathways could also be involved in compensating for the absence of SA in *NahG* plants, which is consistent with their roles in defence against necrotrophic pathogens [40,41]. In this regard, ethylene production has been described to be dramatically increased in CEVd-infected *NahG* plants, pointing to the role of this phytohormone in viroid infections [15]. The distinct VOC profile of *NahG* plants reflects stress-induced metabolic reprogramming due to impaired defence, forcing the plant to adopt less specific defence mechanisms [4,42,43]. Interestingly, elevated levels of benzenoids such as guaiacol and eugenol were observed. Catechol detoxification through methylation likely explains guaiacol accumulation [15,44], while eugenol has been described as a priming agent enhancing virus resistance by stimulating SA and nitric oxide levels, as well as the expression of *SlPer1* resistance genes [45,46]. In any case, VOCs forming part of the so-called “aroma of death” could be used as biomarkers of the viroid disease, since they are associated with viroid symptomatology.

In contrast, *RNAi_S5H* plants, which accumulate elevated SA levels due to the silencing of the SA hydroxylase enzyme (S5H), displayed a restrained metabolic response to CEVd infection. This was supported by PC2 of the PCA (Figure 2), with fewer differential VOCs detected, particularly among key terpenoids known for their signalling roles (Figure S1C, Figure 3, column 3). The limited metabolic shift observed in *RNAi_S5H* plants reflects an SA-primed state that enables a rapid yet targeted defence response, minimizing resource expenditure and metabolic disruption [36,47]. These findings align with previous work which demonstrated that enhanced SA accumulation increased CEVd resistance through glycosylated SA [9].

Wild-type plants exhibited an intermediate response to CEVd infection, characterized by moderate changes in VOCs, including *p*-cymene and methyl salicylate (MeSA) (Table 1). This balanced metabolic profile suggests that wild-type plants activate both SA-dependent and SA-independent pathways, enabling a defence mechanism that minimizes disruption to overall metabolism. The ability to engage multiple pathways, while maintaining metabolic stability, likely underpins their natural CEVd resistance and supports the evidence on SA-JA crosstalk in balancing responses against diverse pathogens [48]. This classic crosstalk between SA and JA was confirmed in *RNAi_S5H* plants, which showed not only enhanced SA production but also compromised JA signalling, displaying susceptibility to *Botrytis cinerea* [9]. Recently, a novel metabolic crosstalk between SA and HMTPs has also been described, due to competition between the MEP pathway involved in the HMTP production and SA biosynthesis [30]. These findings highlight the intricate interplay between hormonal signalling and metabolic pathways, underscoring the complexity of plant defence mechanisms against diverse pathogens.

The specific comparison of VOC profiles from *NahG* vs. *RNAi_S5H* genotypes (Figure 5) revealed that *NahG* plants were characterized by a predominance of monoterpenoids, emerging as potential indicators of plant disease. Terpenes are well documented for their antimicrobial properties and their roles in activating both localized and systemic plant defence responses [20]. Notably, β-myrcene has been described as an antifungal agent against *Fusarium* [49] and an activator of defence responses in rice [50]. Moreover, in tomato plants, monoterpenoids have been described to activate the defence response by inducing stomatal closure and pathogenesis-related protein 1 (PR1) expression [30]. Additionally, our findings align with the role of herbivore-induced volatiles (HIPVs), such as (*E*)-β-ocimene, in enhancing inter-plant defences against pathogens and herbivores [51]. On the other hand, *RNAi_S5H* plants exhibited higher levels of benzenoids, uncovering the MeSA differential emission, and aldehyde derivatives of fatty acids, including (E)-3-hexenal, (E)-2-hexenal, and hexanal. These aldehydes belong to the green leaf volatile (GLV) family, which has been described to have an important role in the activation of plant immunity [28,29]. As for MeSA, it is also involved in the plant defence response against pathogens and herbivores, playing a crucial role in plant-to-plant communication [16,51]. Future research could focus on performing targeted qRT-PCR analyses of genes participating in the biosynthesis of these key VOCs, adjusting sampling times to capture dynamic changes in their expression during infection. Such studies would enhance our understanding of VOC production under stress and support strategies for improving plant resilience through targeted metabolic engineering.

This study demonstrates that CEVd infection triggers genotype-dependent metabolic changes in VOC production, which is largely influenced by SA accumulation levels. SA-deficient plants exhibited broader metabolic shifts, while SA-accumulating plants displayed more targeted and efficient defence responses.

The identification of key VOCs as markers of pathogen tolerance could offer valuable insights for breeding programmes and targeted metabolic engineering, enabling the development of more resilient crops with enhanced defence mechanisms. Specifically, we propose integrating CRISPR/Cas9 or RNA interference to modulate SA levels or regulate VOC biosynthesis pathways. These strategies could be used to optimize the production of these key defence-related VOCs in plants. Our findings contribute to a deeper understanding of plant–pathogen interactions and pave the way for the development of disease-resistant crop genotypes through metabolic and genetic optimization. By leveraging genotype-specific VOC signatures, future strategies can integrate metabolic traits into sustainable disease management and crop improvement efforts.

## 5. Conclusions

Our findings reveal that CEVd infection induces genotypic and salicylic acid (SA)-dependent changes in the VOC production of tomato plants. SA plays a pivotal role in modulating these responses, with SA-deficient plants (*NahG*) undergoing extensive metabolic reprogramming, whereas SA-accumulating plants (*RNAi_S5H*) exhibit a more regulated and targeted defence response.

Notably, the identification of specific VOCs as differentially regulated across genotypes underscores their potential for the development of VOC-based strategies for sustainable disease management. Moreover, the identification of VOCs associated with viroid symptomatology holds promise for the use of these biomarkers for early detection. These findings provide valuable insights into the intricate metabolic interactions shaping plant–pathogen dynamics and offer promising avenues for enhancing crop resistance through targeted metabolic engineering and breeding strategies.

## Figures and Tables

**Table 1:** List of differentially accumulated VOCs in CEVd-infected MoneyMaker tomato leaves.

Compound	Family Code/Number	Retention Time (min)	Specific Ion (*m*/*z)*	Log 2 (CEVd/Mock)
Malonamic acid *	Acid/1	7.98	43	−1.72
(*Z*)-2-Caren-4-ol ^a^*	Alc/1	24.58	107	−2.56
*p*-Cymene	Mt hd/1	24.75	119	1.69
(*E,E*)-Cosmene *	Mt hd/2	24.79	98	−1.48
(*E*)-2-Octenal	Ald/3	25.62	83	1.25
Guaiacol ^b^	Alc/4	26.82	59	1.06
Linalool ^a^	Alc/5	26.99	93	−3.83
Methyl salicylate ^b^	Est/1	30.57	65	1.01

* Tentative identification based on mass spectrum. Family Code: Alc, alcohol; Ald, aldehyde; Est, ester; Mt hd, monoterpene hydrocarbon; Sqt, sesquiterpene. a, monoterpene-derived compound. b, benzene-derived compound.

## Data Availability

The data presented in this study are available in this article and Supplementary Materials.

## Supplementary Materials

The following supporting information can be downloaded at https://www.mdpi.com/article/10.3390/metabo15020102/s1: Supplemental Figure S1: Volcano plots showing differentially expressed volatile organic compounds (VOCs) in *Solanum lycopersicum* genotypes (wild-type, *NahG*, and *RNAi_S5H*) upon Citrus Exocortis Viroid (CEVd) infection compared to mock treatment. Each dot represents a VOC, plotted by its statistical significance (−log_10_ *p*-value) against its fold change (log_2_FC). Compounds with high fold changes are positioned toward the sides of each plot, indicating strong over- or under-accumulation in response to CEVd infection. Colours represent the fold-change magnitude, with red indicating high over-accumulation and blue indicating high under-accumulation. Supplemental Table S1. Retention times and characteristic mass values for volatile organic compounds (VOCs) identified in Solanum lycopersicum genotypes. Compound retention time (in minutes) and primary mass-to-charge ratio (m/z) were determined using gas chromatography–mass spectrometry (GC-MS). Compounds with asterisks (*) indicate tentative identification.

## Abbreviations

The following abbreviations are used in this manuscript:

CEVd	Citrus Exocortis Viroid
SA	salicylic acid
GC-MS	gas chromatography–mass spectrometry
VOCs	volatile organic compounds
