# Supplementary material for: Salicylic Acid Modulates Volatile Organic Compound Profiles During CEVd Infection in Tomato Plants
Source: Metabolites. 2025 Feb 6;15(2):102. doi: 10.3390/metabo15020102 (PMC11857198; doi:10.3390/metabo15020102)
Supplement: Supplementary file 1 [file metabolites-15-00102-s001.zip › metabolites-3421874-supplementary.pdf]

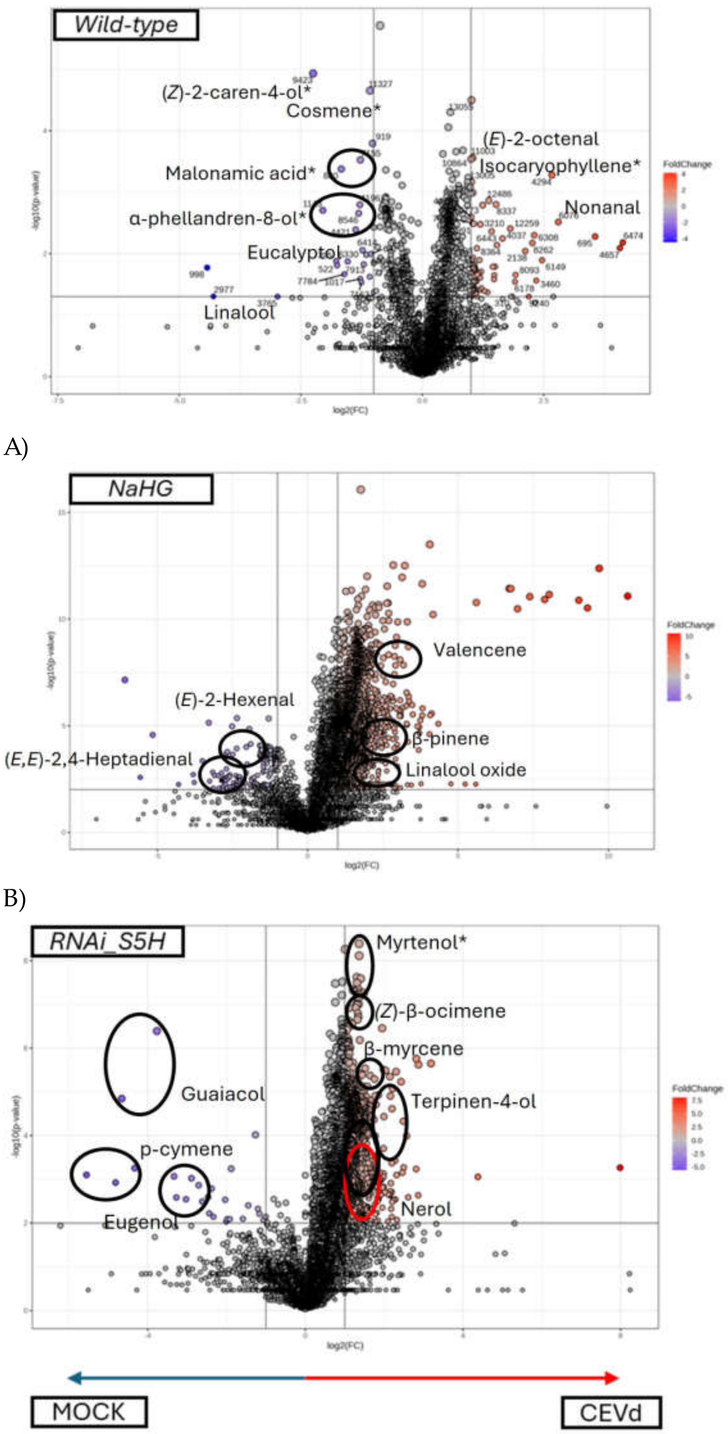

C)

Supplemental Figure S1. Volcano plots showing differentially expressed volatile organic compounds (VOCs) in *Solanum lycopersicum* genotypes (Wild-type, *NahG* and *RNAi_S5H*) upon Citrus Exocortis Viroid (CEVd) infection compared to MOCK treatment. Each dot represents a VOC, plotted by its statistical significance ($-\log_{10}$ *p*-value) against its fold-change ($\log_2$FC). Compounds with high fold-changes are positioned toward the sides of each plot, indicating strong over- or under-accumulation in response to CEVd infection. Colours represent the fold-change magnitude, with red indicating high over-accumulation and blue indicating high under-accumulation.

Supplemental Table S1. Retention times and characteristic mass values for volatile organic compounds (VOCs) identified in *Solanum lycopersicum* genotypes. Compound retention time (in minutes) and primary mass-to-charge ratio (*m/z*) were determined using gas chromatography-mass spectrometry (GC-MS). Compounds with asterisk (*) indicates tentative identification.

**Compound**	**Retention time (min)**	**Mass (*m/z*)**
Malonamic acid *	7.98	43
(*E*)-2-Hexenal	18.05	80
β-Myrcene	23.12	57
β-Pinene	23.31	106
Octanal	23.61	84
(*E,E*)-2,4-Heptadienal	24.06	81
3-carene	24.28	91
(*Z*)-2-Caren-4-ol *	24.58	107
(*Z*)-β-ocymene	24.71	137
p-Cymene	24.73	119
(*E,E*)-Cosmene*	24.79	98
D-limonene	24.94	72
Eucalyptol	25.22	125
(*E*)-2-Octenal	25.62	83
γ-Terpinene	25.87	57
(*Z*)-Linalool oxide	26.31	59
Guaiacol	26.82	81
Terpinolene	26.89	93
Linalool	26.98	93
Nonanal	27.14	57
Myrtenol	29.21	79
α-Phellandrene-8-ol *	29.50	44
α-Terpinen-4-ol	30.20	71
α-Terpineol	30.56	59
Methyl Salicylate	30.57	65
Nerol	31.10	55
Geraniol	31.76	41
Eugenol	35.22	164
Isocaryophyllene*	37.19	41
α-Humulene	38.66	80
β-Selinene *	39.55	105
Valencene	39.63	161
(*E*)-Nerolidol	40.45	93
